# A Behaviour Monitoring System (BMS) for Ambient Assisted Living

**DOI:** 10.3390/s17091946

**Published:** 2017-08-24

**Authors:** Samih Eisa, Adriano Moreira

**Affiliations:** Algoritmi Research Centre, University of Minho, 4800-058 Guimarães, Portugal; adriano.moreira@algoritmi.uminho.pt

**Keywords:** in-house monitoring of older adults, Ambient Assisted Living, learning mobility routine at home, detecting abnormal behaviour

## Abstract

Unusual changes in the regular daily mobility routine of an elderly person at home can be an indicator or early symptom of developing health problems. Sensor technology can be utilised to complement the traditional healthcare systems to gain a more detailed view of the daily mobility of a person at home when performing everyday tasks. We hypothesise that data collected from low-cost sensors such as presence and occupancy sensors can be analysed to provide insights on the daily mobility habits of the elderly living alone at home and to detect routine changes. We validate this hypothesis by designing a system that automatically learns the daily room-to-room transitions and permanence habits in each room at each time of the day and generates alarm notifications when deviations are detected. We present an algorithm to process the sensors’ data streams and compute sensor-driven features that describe the daily mobility routine of the elderly as part of the developed Behaviour Monitoring System (BMS). We are able to achieve low detection delay with confirmation time that is high enough to convey the detection of a set of common abnormal situations. We illustrate and evaluate BMS with synthetic data, generated by a developed data generator that was designed to mimic different user’s mobility profiles at home, and also with a real-life dataset collected from prior research work. Results indicate BMS detects several mobility changes that can be symptoms of common health problems. The proposed system is a useful approach for learning the mobility habits at the home environment, with the potential to detect behaviour changes that occur due to health problems, and therefore, motivating progress toward behaviour monitoring and elder’s care.

## 1. Introduction

The problem of detecting unusual changes in the daily behaviour of an elderly person who lives independently at home has been widely investigated in the literature [1]. Solutions typically are sensor-based systems that require the use of wearable and non-wearable sensors to track the daily behaviour and provide responses when deviations are detected. These solutions usually require the intervention of the resident user, for instance, by pushing a button on a pendant or on a wrist watch, or by monitoring the resident using camera-based sensors installed at different locations in the house. However, prior research studies [2] show that wearable and camera-based sensors are not very appreciated by the elderly people due to inconvenience, are computationally complex, and raise privacy issues. The elderly might not feel comfortable wearing sensors all the time and may forget to wear them on some occasions, or may feel they are losing their privacy when being monitored by cameras at home. This reduces the usefulness of these sensors for continuous behavioural monitoring. Even though some recent research studies propose including the sensors in people’s clothes [3] or utilising the capabilities of smart watches [4] for behaviour monitoring, these works are still limited and not affordable for everyone, besides the limitations of the sensors’ battery energy. Moreover, most of the existing systems entail an explicit annotation or labelling process to be made offline in order to manually configure the typical behaviour of the monitored persons before using the system, which increases the required installation time of these systems and prevents them from being adaptive to small shifts in behaviour that do not necessarily represent unusual behaviour (e.g., seasonal changes).

In this research work, we are interested in detecting unusual changes in the regular mobility behaviour of independently living older adults by monitoring their daily navigations between rooms at home. In this paper, we describe how we approach this goal by designing a system that automatically learns the daily room-to-room transitions and permanence habits in each room at each time of the day and generates alarm notifications when deviations are detected. We hypothesise that the relationship between changes in behaviour can be observed using data collected from smart home sensors. Hence, our system uses passive infrared (PIR) motion sensors as primary input to track the mobility of the monitored person and also is designed to be agnostic of the user’s daily mobility profile. No explicit annotation or laborious labelling is required to manually configure the mobility profile of the monitored person or to train the underlying behavioural model. However, our approach is used mainly for monitoring the behaviour of a single user living alone at home, it does not take into account the presence of external people at home when leaning the behavioural model of the monitored person.

## 2. Monitoring Human Behaviour

Monitoring human behaviour is not a trivial task. In fact, there is no single approach that claims to cover all aspects of human behaviour monitoring. However, with the recent advent of sensing and communication technologies, many innovative approaches have emerged and quite promising results have been achieved. In this section, a brief description on the topic of human behaviour monitoring is given, with more focus on technologies used for behaviour monitoring for Ambient Assisted Living (AAL) in smart home environments.

### 2.1. Monitoring Activities of Daily Living (ADLs)

Most of the existing Ambient Assisted Living (AAL) systems for elders’ care exploit the activity that is being performed by the elderly at home as a means for inferring or assessing the functional health status of the elderly. An elderly person remains in good health as long as he or she can carry on his/her daily activities as usual with no significant deviations from the normal daily routine. The Activities of Daily Living (ADLs) in the home environment can be categorised into two main categories: basic ADLs (e.g., personal hygiene, bathing, eating, dressing, and functional mobility) and instrumented ADLs (e.g., cooking and housework) [1,5]. Monitoring ADLs of the older adults allows healthcare providers to continuously monitor the functional status of the elderly, increases their ability to live independently, and allows for early detection of diseases such as Alzheimer’s [6,7], dementia [8,9,10] and urinary tract infection [11]. ADLs also can be used to learn daily behaviour patterns such as sleeping habits [12] or movement patterns [13].

#### 2.1.1. Sensing Technologies

In general, there are two main categories of sensors used for monitoring human behaviour: wearable and non-wearable sensors [5]. The two kinds of sensors have been used extensively in various systems. Wearable sensors used for activity recognition and ADLs classification vary depending on the nature of the required application. They are usually attached to the human body or clothes or can be part of or make use of devices that people usually carry with them, such as wristwatches or cell phones. However, accelerometers are the most commonly used wearable sensors with a variety of applications and usage scenarios. Accelerometers are used to identify the location of a person and differentiate motions (e.g., running, walking, walking upstairs, cycling, etc.) [14] or to detect falls using acceleration data from wristwatches [15] or to classify posture of a person by monitoring the tilt of certain parts of the body using the acceleration due to gravity [1]. Moreover, accelerometers are combined with gyroscopes to obtained orientation information [16] and also with tilt switches in a wrist-worn unit sensor [17] to capture the user’s behaviour rhythms in day-to-day activities as a way to improve long-term activity recognition.

Smartphones are also considered wearable sensors. They are equipped with multiple sensors that provide a wealth of information for different applications (e.g., global positioning system (GPS), cameras, microphones, light, temperature, magnetic compasses, gyroscopes, and accelerometers). In [18], a framework that exploits the rich contextual information from smartphones (e.g., location, time, apps, call logs, and internal state) is presented. The framework uses the data collected from smartphone sensors to predict our next destination and which app we will be using in the next ten minutes. In [19], cell phone accelerometers are used for recognising activities such as walking, running, and jogging.

In addition, other kinds of wearable sensors also have been used for ADLs monitoring such as magnetic sensors [20] for monitoring activities and use of portable devices, RFID sensors for detecting interactions with objects in smart homes and recognizing activities like cooking, washing dishes [21], teeth brushing and watching TV [22], and inertial sensors [23] for providing assessment of patient progress after an injury or stroke in ecological rehabilitation environments. Besides the use of wearable sensors to monitor clinical measurements or vital signs such as pulse rate, body humidity and temperature, respiration rate, and blood pressure [3,24]. However, the power consumption requirement and the convenience of constantly wearing wearable sensors are the major challenges that face the use of these sensors for long-term human behaviour monitoring.

Non-wearable sensors also are being used for ADLs monitoring [25,26,27]. Infrared (IR) sensors are the most commonly used non-wearable sensors. They are used for detecting presence, motion or locating people at home. In [10], passive infrared (PIR) sensors were used for managing dementia and depression diseases. The collected data from PIR sensors were used for the early detection of changes in activity level which was used to reduce the advancements of these diseases and lead to early interventions. Additionally, other non-wearable sensors are also being used for ADLs monitoring such as ultrasonic sensors [28], pressure sensors [29], vibration sensors [30], video-based sensors, low-resolution thermal sensors [31], wattmeter sensors [32], water flow sensors [33], magnetic door switches [34], and audio or sound sensors [35]. Table 1 presents a comparison of some properties of a set of sensors that were explored for ADLs detection.

#### 2.1.2. Activity Classification

Many data mining and machine learning algorithms are used for ADLs classification: support vector machines (SVM) [36,37], random forest [38], decision trees [20], fuzzy logic [39], Bayesian methods [40], and neural networks [37]. However, most of these classical machine learning algorithms assume that the input data for the classification step is independent and identically distributed (IID). This assumption does not hold in the case of human behaviour modelling and recognition, what a person is doing at the moment is not independent of what he was doing just before. Hence, more advanced models are required to handle the case when IID does not hold. In [5], two main categories that consider the dependency assumption are defined: generative approaches (e.g., hidden Markov model (HMM) [41]) and discriminative approaches (e.g., conditional random field (CRF) [15]). Many algorithms from these two categories have been used widely and successfully in many behaviour modelling and ADLs classification applications. However, a previous intensive supervised training stage is required to estimate their models, which in turns may lead to human bias when labelling and annotating the activities.

Statistical (heuristic) approaches are also being used for human behaviour monitoring [25,42]. A system that examines the activity rhythms at home using a statistical predictive algorithm is presented in [25] to evaluate the behaviour of a resident individual while performing the daily activity routine. In some other cases, simple statistics (heuristics) measures are used as features for a second-level activity classification algorithm, as implemented in [43], where heuristic measures like means and variances were used as features for neural network models to detect and classify motion activities.

### 2.2. Monitoring Location Context

The location of a person is essential for measuring the activity and assessing the overall behaviour of the person. The term location includes both indoor and outdoor locations.

#### 2.2.1. Indoor Location

The indoor location of a person at home gives useful information to build behavioural models for his everyday life. The movements of people inside the home correlate to their daily physical activities and performance of the activities of daily living. For example, frequent visits to the bathroom at night during sleeping time can be an indicator for significant sleep disorder or nocturia disease and may be a sign of developing urinary tract infections disease [44]. These kinds of unusual sleep disorders can be detected by a localisation system that continuously monitors the location of a person at home. In [11], an integrated sensor network of PIR motion sensors, bed and chair sensors, was used in apartments of volunteer residents at an ageing in place retirement community. The sensors were used for detecting pulse, respiration rate and bed restlessness which, in turn, were used for detecting urinary tract infections. PIR motion sensors also were used in [45] to determine the location of an older adult in a smart home environment and to infer the activities of daily living (ADLs) such as sleeping, preparing meals, going out, toileting, and brushing. In [46], an ultra-wide-band (UWB) location system (UBISENSE) was used for monitoring the elderly people suffering from dementia, and in [47], an ultrasonic indoor location tracking system also was used for enabling location-aware pervasive service to monitor the older adults at home.

#### 2.2.2. Outdoor Location

The outdoor mobility of the elderly is also important for evaluating their quality of life when going outside. Elderly people who have physical mobility limitations tend to have a lower quality of life and involvement in social communities [48]. In [49], a disorientation detection method that detects outliers in a person GPS mobility trajectories is presented. A survey for mining GPS data for mobility patterns is presented in [50]. In this paper, we mainly focus on behaviour monitoring at home environment and, therefore, we will not dive further into behaviour monitoring using outdoor location. We do, however, consider in our system the modelling of the time periods when the persons leave their homes.

### 2.3. Detection of Abnormal Behaviour

Abnormal behaviour refers to finding unexpected behaviours that do not conform to usual behavioural routines [51]. This topic has been investigated widely and applied in many domains and application scenarios. The detection of abnormal human behaviour depends on the way the human behaviour is defined. Activity recognition is the main approach for abnormal human behaviour detection [52]. The deviations in the activities of daily living (ADLs) are considered the most common way of defining abnormalities in the human behaviour. By monitoring the performed daily activities of a person for a certain time, one can learn and build a model of normal behaviour and then detect deviations. In other work [42], an abnormal human behaviour was defined as an increase or decrease in the daily physical activity, defined as any body movement produced by skeletal muscles that result in energy expenditure. Deviation or change in the physical activity level, according to historical data, was used as an early symptom of health problems. In general, the abnormalities in the human behaviour are mainly related to the detection of falls [53,54], inactive periods [55,56], abnormal living patterns [57], long-term behaviour changes [24], or abnormal events such as sleeping disorder [58].

## 3. Behaviour Monitoring System (BMS)

**Figure 1:**
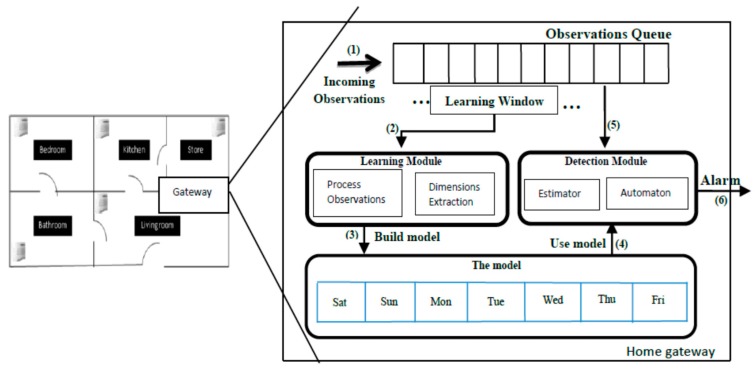
BMS system architecture.

The general architecture of the BMS is graphically represented in Figure 1. A set of sensors is placed at different locations to collect information about a person’s daily mobility routine at the home environment. The collected data from the sensors is forwarded (1) to a home gateway installed inside the house that continuously processes and interprets the data locally, learns the normal routine of the person (Learning Module and Model) and then provides alarm notifications (6) when unusual deviations from the normal routine are detected (Detection Module).

### 3.1. Learning Module

The deployed PIR sensors in the home environment transmit signals when motion is detected. These sensor observations are logged in the home gateway and stored in a queue buffer for processing. An observation o is defined in the form o = <ts, sensor_id>, where ts denotes a timestamp indicating the time of detection, and sensor id denotes the identification of the sensor that detected the motion. Each sensor is assumed to be installed in a particular room in the house, and each room must have at least one sensor covering its serving area. The operation of the system relies on the detection of the monitored person within the range of these sensors with room-level localisation accuracy.

We hypothesise that a long-term sequence of these observations encodes the mobility routine of the monitored person, and therefore it can be used to build a model that represents the mobility behaviour of that person during normal days. The role of the learning module in our system is to continuously process and interpret the incoming sensor observations and use them to build a realistic model that summarises the mobility behaviour of the monitored person at each location in the house during the hours of the day. The learning module uses a time-based sliding window to process the observations from the queue buffer sequentially. The size of the learning window is pre-configured (e.g., a month) to indicate the sufficient context history to tune the model. The learning window is shifted every week (the shift of one week is related to the structure of the model, as described in Section 3.2.) to update the model on a weekly basis. Every time the model is updated, the oldest observations are removed from consideration, while the most recent observations are added. This feature allows the learning process to be performed in an online manner, considering the most recent observations, and also allows the model to adapt to slight shifts in behaviour that are not genuine anomalies (e.g., seasonal changes).

### 3.2. The Model 

**Figure 2:**
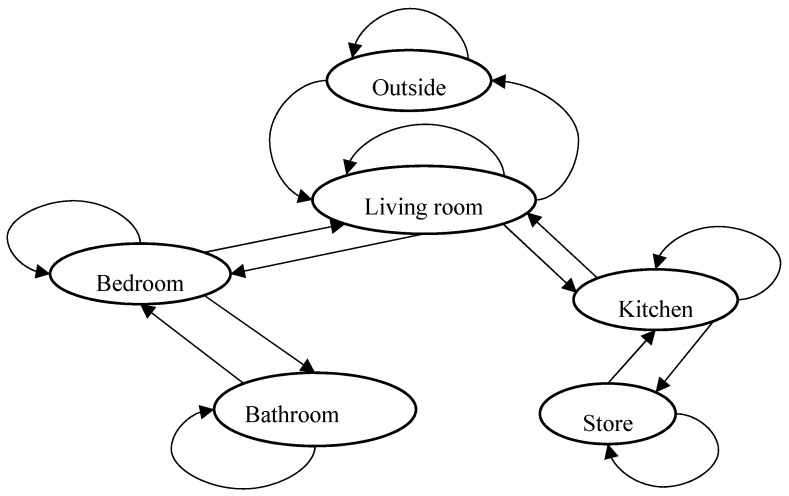
Room-to-room State Transition Model.

To model the daily mobility behaviour, we define the concept of Stay to indicate the amount of time a person has spent in a particular room and time. A Stay is obtained from any pair of consecutive sensors observations (o_i−1_, o_i_). It is defined as the time elapsed between o_i−1_ and o_i_ at the room associated to o_i−1_. The Stay concept is fundamental for the learning module to estimate the dimensions of the underlying behavioural model. As shown in Figure 1, the model is represented by a data structure that separates the mobility behaviour of the monitored person in each day of the week to capture the weekly behaviour. Furthermore, each day is subdivided into equal intervals (e.g., 1-h interval) to capture the daily behaviour. This flexible structure differentiates our work from others where the same behaviour is assumed for all the weekdays. In each interval, the mobility profile of the monitored person is represented by a state transition model, as shown in Figure 2. The states represent the rooms or places in the home while the connections between states represent the possible transitions or movements. This representation is used to model the spatial transition dependencies between the rooms with respect to the layout of the user’s house. As illustrated in Figure 2, there are no transitions between rooms that are not directly linked (e.g., one cannot go directly from the bedroom to the kitchen without going through the living room). By observing the daily movements from sensor observations and then applying the right learning method we can build a reliable model for the daily mobility behaviour of the monitored person and use that model to detect abnormalities. The model can be formulated as Model^d^ (t, ∆t) to denote the model of day d in the time interval from t to t + ∆t, d = {Sunday…, Saturday}. For example, if ∆t = 1 h then there will be 24 time intervals and models in each day: Model^Sunday^ (0–1], Model^Sunday^ (1–2], …, Model^Saturday^ (23–24].

A model for a given time interval is defined by a set of dimensions, each dimension has its own unique meaning and contribution in describing the person’s behaviour. The following subsections describe these dimensions. It is worth mentioning that our model considers the person to be staying at the location reported by an observation until a new observation indicates the presence at a different location. This assumption is related to the typical operation of the PIR sensors, since they are usually triggered when they detect movement, and then enter a sleeping mode for some time to preserve the energy of their batteries.

#### 3.2.1. Transition Matrix

This is the fundamental dimension of the behavioural model. It is a direct and personalised representation of the daily stay and room-to-room transition behaviour of the monitored person. It shows how likely is to find the monitored person in each room during the different hours of each day of the week, considering the differences in every person’s habits. Each possible transition in the state model (Figure 2) has an associated probability P^i,j^ representing the estimated probability of that transition. All transition probabilities between rooms for a given time interval, including self-transitions, are represented in a 2D matrix. The role of the learning module here is to compute the entries of this transition matrix by processing the entire observations in the learning window, computing the stay durations between any pair of consecutive observations, calculating the total stay time at each room within each time interval, and finally computing the transition probabilities. These steps are implemented as follows. In the examples below, and without loss of generality, we consider ∆t = 1 h.

Step 1: Compute a stay duration

A pair of consecutive observations in the queue buffer (o_1_ = <ts_1_, sensor_id>, o_2_ = <ts_2_, sensor_id>) represents a stay(s) in the model. The duration of this stay is calculated as the time elapsed between these two observations (ts_2_ − ts_1_). It may happen that a single stay overlaps with multiple intervals in the model. In this case, we distribute the period among them according to their contributions in the total stay period. For example, a stay duration of 1 h and 30 min that starts at ts_1_ = 04:00 and ends at ts_2_ = 05:30 is distributed among two intervals. In this case, 1 h will be assigned to the interval (4–5], and the remaining 30 min to the interval (5–6].

Step 2: Compute total stay time

The total stay time TS^i^ (t, ∆t) at a room within a given time interval is given by:

(1) $${\mathrm{TS}}^{i}\left(t,\;\Delta t\right)=\overset{{\mathrm{ns}}^{i}}{\underset{k=1}{\sum }}s_{k}^{i}\left(t,\;\Delta t\right)$$

where s_k_^i^ (t, ∆t) denotes the kth stay at room i, and ns^i^ denotes the number of stays at room i within the time interval from t to t + ∆t.

Step 3: Compute self-transition, or stay probability

The self-transition probability P^i,i^ (t, ∆t) at a room ***i*** in a given time interval represents the probability of finding the person at that room (i) within that time interval (from t to t + ∆t) and is given by:

(2) $$P^{i,i}\left(t,\;\Delta t\right)=\frac{{\mathrm{TS}}^{i}\left(t,\;\Delta t\right)}{\sum_{j=1}^{r}{\mathrm{TS}}^{j}\left(t,\;\Delta t\right)}=\frac{{\mathrm{TS}}^{i}\left(t,\;\Delta t\right)}{\Delta t}$$

where r denotes the number of rooms in the house, including a virtual room for outside of the house.

Step 4: Compute transition probability

The transition probability P^i,j^ (t, ∆t) within a given time interval (from t to t + ∆t) represents the probability of observing a person’s movement from room ***i*** to room ***j*** within that interval. Given that:

$$\sum_{j=1}^{r}P^{i,j}\left(t,\;\Delta t\right)=1$$

$P^{i,j\;}\left(t,\Delta t\right)$ is given by:

(3) $$P^{i,j}\left(t,\;\Delta t\right)=\left[{1-P}^{i,i}\left(t,\;\Delta t\right)\right]\times \frac{M^{i,j}\left(t,\;\Delta t\right)}{\sum_{k=1\;k\neq i}^{r}M^{i,k}\left(t,\;\Delta t\right)}$$

with j ≠ i and M^i,j^ (t, ∆t) denotes the number of transitions from room i to room j in the time interval from t to t + ∆t.

The following is an example of a complete transition matrix for the six-rooms house in Figure 1. The entries of the matrix indicate the calculated room-to-room transition probabilities for a given time interval:

$$\left[\begin{array}{ccccccc} & \text{Bedroom} & \text{Bathroom} & \text{Livingroom} & \text{Kitchen} & \text{Store} & \text{Outside}\\ \text{Bedroom} & 0.1 & 0.3 & 0.4 & 0.2 & 0 & 0\\ \text{Bathroom} & 0.3 & 0.7 & 0 & 0 & 0 & 0\\ \text{Livingroom} & 0.2 & 0 & 0.3 & 0.2 & 0 & 0.3\\ \text{Kitchen} & 0 & 0 & 0.3 & 0.5 & 0.2 & 0\\ \text{Store} & 0 & 0 & 0 & 0.9 & 0.1 & 0\\ \text{Outside} & 0 & 0 & 0.8 & 0 & 0 & 0.2 \end{array}\right]$$

The estimated values of the matrix indicate how probable this person tends to stay or navigate between the rooms of the house. The matrix is not symmetric so that M^i,j^ ≠ M^j,i^ and 0 probability indicates no transition was detected.

#### 3.2.2. Global Activity (AG)

This is an accumulative dimension to count the total number of observations received within an interval, no matter the rooms in which the sensors were triggered, normalised to the length of the time interval. It models the level of activity within the house, as more activities (movements) translate into more sensors being triggered. The following Equation (4) is used to compute this dimension:

(4) $$AG\left(t,\Delta t\right)=\frac{1}{\Delta t}\times \#\left(O\left(t,\Delta t\right)\right)$$

where #(O(t, ∆t)) denotes the cardinality of the received sensors’ observations within the time interval from t to t + ∆t.

#### 3.2.3. Inter-Room Activity (AE)

The Inter-room Activity dimension represents the total number of times a sensor was triggered to indicate a transition within an interval, excluding self-transitions. It represents how often a person moves between rooms within a given time interval. This is different from the Global Activity because it captures the transitions among different rooms, while the Global Activity dimension captures the activities that might be within a single room. The following Equation (5) is used to compute this dimension:

(5) $$\mathrm{AE}\left(t,\;\Delta t\right)=\frac{1}{\Delta t}\times \#\left(M\left(t,\;\Delta t\right)\right)-\overset{r}{\underset{i=1}{\sum }}\#\left(M^{i,i}\left(t,\;\Delta t\right)\right)$$

where # (M (t, ∆t)) denotes the cardinality of all the transitions within the time interval from t to t + ∆t and # (M^i,i^ (t, ∆t)) denotes the cardinality of the self-transitions in room i.

#### 3.2.4. Intra-Room Activity (AA)

The Intra-room Activity dimension represents the total number of self-transitions in a room within an interval, computed as the total number of received observations in a room within an interval. This dimension shows how active the person was in each room. The Intra-room Activity is given by:

(6) $${\mathrm{AA}}^{i}\left(t,\;\Delta t\right)=\frac{1}{\Delta t}\times \#\left(O^{i}\left(t,\;\Delta t\right)\right)$$

where # (O^i^ (t, ∆t)) denotes the cardinality of the received Observations at room i within the time interval from t to t+ ∆t.

#### 3.2.5. Intra-Room Continuous Stay (CS)

This dimension is used to estimate the longest continuous stay at each room in each day of the week. A continuous stay is defined by a sequence of consecutive stays, or a single long stay, that occurs in the same room with no interrupting stay in a different room. The dimension is given by:

(7) $${\mathrm{CS}}^{i}=\overset{u}{\underset{k=1}{\sum }}{d(s}_{k}^{i})$$

where d(s_k_^i^) denotes the duration of the k^th^ stay at room i and u denote the number of consecutive stays in room i. In the model, each day has a list of continuous stay for each room.

### 3.3. Detection Module

**Figure 3:**
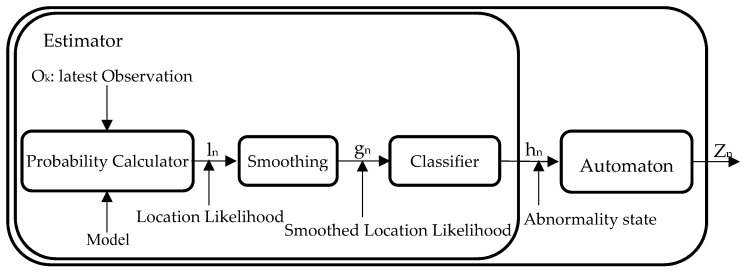
Detection Module—internal structure.

The detection module is a separate process that runs continuously in real time, and asynchronously from the learning module to produce outputs at regular intervals (e.g., every one minute). This module consists of two main components: the estimator and the automaton. The estimator computes the location likelihood for the detected location of the monitored person and provides a classified binary abnormality state. The structure of the detection module is illustrated in Figure 3.

#### 3.3.1. Estimator

In every running cycle of the detection module, the estimator takes the most recent received observation in the queue buffer o_k_ and compares the current stay at the room reported in that observation to what is expected based on the behavioural model created from past observations. This comparison is based on estimating how probable it is to observe the person in that room during this interval and day of the week. For example, consider that, on a Monday at 4:25 a.m., the latest observation o_k_ reported the person as being in the bedroom. Given the behavioural model for the current time interval, the probability of finding the person in that room is 0.97 (Equation (2)) and therefore, the observed behaviour was expected with high probability. We call this probability the Location Likelihood l_n_.

A simplistic way for detecting abnormal behaviour is obtained by just comparing the estimated Location Likelihood l_n_ with a fixed threshold. All estimates higher than the threshold indicate no significant change in the mobility routine, while any drop below the threshold indicates a presence at an abnormal location given the current time and weekday. However, through experimentation, we found that this simple approach is affected by spurious observations and leads to poor performance. Moreover, the fixed threshold approach is also affected by the fact that, in some cases, the most probable location has a probability much lower than 1. Therefore, a more elaborated method is required to improve the reliability of the detection. To deal with this, we added a normalisation step in the probability calculator. The estimated Location Likelihood l_n_ is normalised by the probability of the most probable location of the current time interval before passing through a low-pass filter to smooth out the results and generate smoothed Location Likelihood g_n_ and then finally applying the threshold classifier.

**Figure 4:**
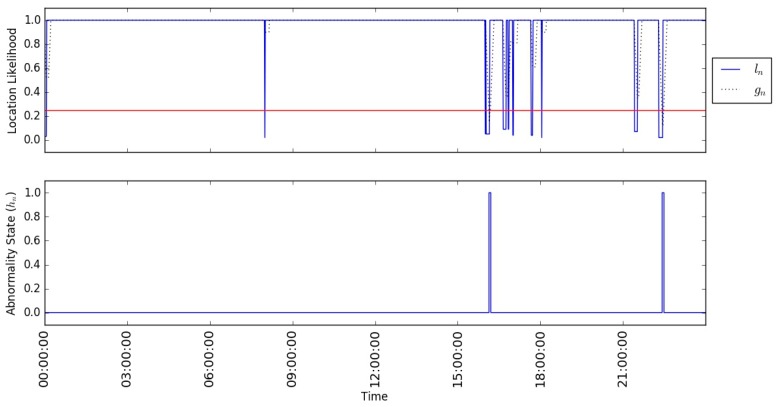
Estimator’s results (smoothed and classified) synthetic dataset (user Profile A).

Figure 4 illustrates an example of the generated estimator’s results using synthetic data (user Profile A). It shows the process of applying the smoothing step on the normalised estimated Location Likelihood l_n_ to smooth out the generated signal and reduce the rate of false alarms (whenever the location likelihood drops below the threshold). The example in the figure shows the estimated results of a normal day so that the estimated Location Likelihood ***l_n_*** should always be high (near to 1.0) and any low value is considered false alarm. As shown in the figure, the smoothing step may reduce the rate of false alarms, however, it does not eliminate them all. The smoothed Location Likelihood g_n_ afterwards is passed through the threshold classifier to generate the classified abnormality state results h_n_, as shown in the figure. The estimator keeps generating abnormality states, on a regular basis, while the observations are arriving, randomly, based on the person’s movement. This way we transform the outputs of the estimator into periodic time series to simplify the detection of the abnormal changes.

#### 3.3.2. Automaton

**Figure 5:**
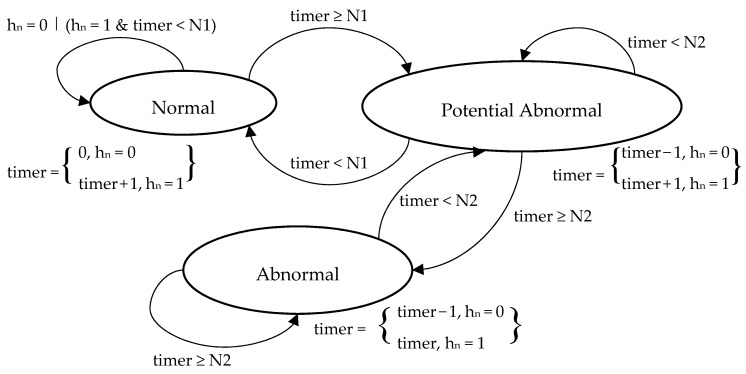
Detection Module—Automaton.

The estimator generates binary classifications (0 = normal or 1 = abnormal) based on the evaluation of the detected location of the monitored person. However, binary outputs are not a very informative feedback to show the real state of the subject and might be misleading and not realistic in some cases. Therefore, it is more convenient to define the final output of the detection module in terms of states, giving more descriptive and detailed explanation of the decisions made. For this, we defined an automaton with three states {Normal, Potential Abnormal, and Abnormal} to interpret the detection results. Figure 5 illustrates the states of the automaton. The state of the automaton is updated at a regular pace defined by the output (h_n_) of the estimator, including one timer that is reset, incremented or decremented.

The normal state represents the case in which the detection module doesn’t detect any significant deviation or shift in the daily routine of the monitored person. The automaton is kept in this state as long as the output of the estimator reports a normal behaviour (h_n_ = 0), or while abnormal outputs (h_n_ = 1) do not hold for more than the normal timeout N1. This way, short glitches of abnormal outputs followed by normal outputs are filtered out, thus reducing the rate of false alarms. The “Potential Abnormal” state represents the cases in which the estimator reports a sequence of abnormal behaviours longer than N1 (meaning that it might not be a glitch anymore), and therefore more attention must be given to the situation. The automaton changes to the “Abnormal” state if the estimator keeps reporting an abnormal state for a long time (t ≥ N2). The difference between the states “Normal” and “Potential Abnormal” is that the timer is not reset after the first normal output of the detection module (h_n_ = 0). The transition to “Abnormal” state can be done only in case the detection timer exceeds the abnormal timeout (N2), indicating the confirmation of the detected abnormal state. Upon each decision, the detection timer is updated according to the rules of each state. The transition constraints between states are shown on the automaton diagram, explaining the rules and conditions that govern each transition. Applying such state machine simplifies the final decision of the detection module, gives more flexibility and also reduces the rate of false positive alarms.

### 3.4. Classification of Abnormal Behaviour

An elderly person may have different kinds of abnormal behaviours when performing everyday activities at home, and it is not trivial to anticipate all of them in advance. Thus, we limited ourselves to a finite set of abnormal behaviours to be detected by our system. Table 2 lists these abnormal behaviours, their descriptions and their related symptoms and possible health-declines.

**Figure 6:**
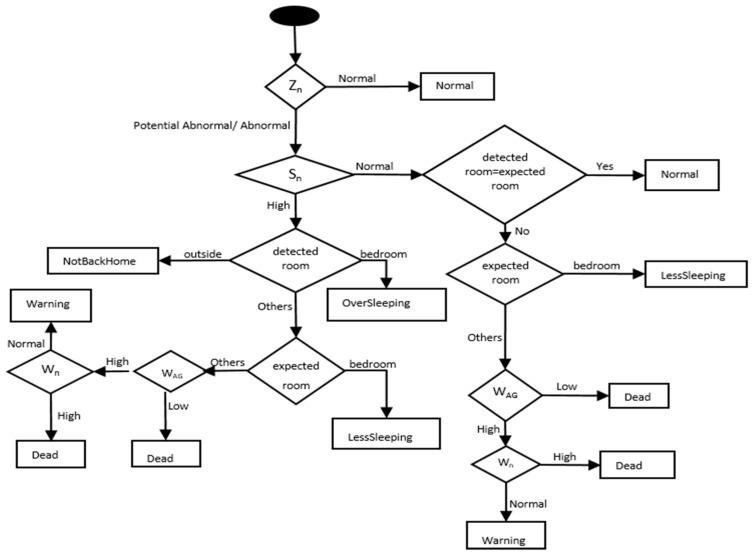
Rule-based Anomaly Classification.

In the detection module, we added an additional step to classify the detected abnormal behaviours so that caregivers and healthcare providers can easily understand and react upon. This was done by introducing a rule-based classifier that incorporates the output of the automaton with the other dimensions of the behavioural model to give classifications for the detected abnormal behaviours. Figure 6 shows the flow chart of the rule-based anomaly classification step.

In the above flow diagram, the following symbols and names are used:

- Z_n_: represents the automaton’s output (Normal, Potential Abnormal, Abnormal).
- S_n_: represents the classification of the detected longest continuous stay (Normal or High). The detected longest stay is “Normal” if its value is less than the expected longest stay value from the model; otherwise, it is “High”.
- Detected room: represents the room where the person was detected.
- Expected room: represents the room where the person is expected to be according to the model.
- *W_AG_*: represents the weighted Global Activity. It is computed based on the current and previous time intervals from the model (Equation (8)).
- W_n_: represents the classification of the weighted Global Activity (W_AG_). The individual classification of the W_AG_ and the sampled global activity (S_AG_) at the current time. The table below (Table 3) shows the matching matrix used to come up with the final classification for W_n_. The sign √ means Normal and 〤 means Abnormal. The values in the matrix are based on how closed W_AG_ and S_AG_ are to the max Global activity from the mode:
  (8) $$W_{\mathrm{AG}}=\frac{\mathrm{minutes}}{\Delta t}{\times \mathrm{AG}}_{\mathrm{model}\;(\mathrm{current}\;\mathrm{interval})}+\frac{\Delta t-\mathrm{minutes}}{\Delta t}{\times \mathrm{AG}}_{\mathrm{model}\;(\mathrm{previous}\;\mathrm{interval})}$$
  where minutes denotes the elapsed time in the current time interval (in minutes) and Δt denotes the length of the time interval (e.g., 60 min).

## 4. Experiments

We performed several experiments to validate the developed BMS. In this section, we present the descriptions of the datasets used for validation and the settings of the experiments.

### 4.1. Datasets

Two different types of datasets have been used for validation, named the Synthetic and the Aruba datasets. Table 4 presents a summary of the datasets.

#### 4.1.1. Synthetic

A synthetic data generator was developed to simulate different user’s mobility profiles at home. For the experiments in this paper, we generated two different user profiles (Table 5). The first user profile for a morning person (Profile A) and the second profile for a nightly person (Profile B). The simulated behaviours include three different periods of the day with different behaviours: sleeping at the bedroom, begin out of the home, and staying in the living room. The two user profiles are extremely different from each other to show the ability to learn different users’ profiles. The layout of the home is shown in Figure 1; with a virtual “outside” room to model the periods when the person goes outside of the home.

#### 4.1.2. Aruba

**Figure 7:**
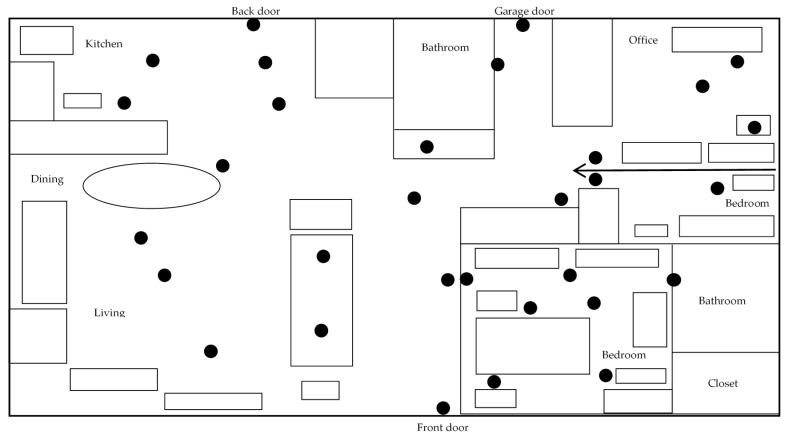
The Aruba home layout; adapted from [59].

The Aruba dataset was collected from the CASAS Smart Home Project at Washington State University (WSU) (Pullman, WA, USA, http://casas.wsu.edu/datasets/). It contains sensors data that was collected in a home of a volunteer adult woman. Figure 7 shows the layout of the home. In our experiments, we consider observations collected from the PIR motion sensors only and ignore the other type of sensors. The locations of the motion sensors are shown in the home’s layout (dark circles).

### 4.2. Settings

Table 6 presents the experiments settings, showing the experimentally optimised parameters of the learning and the detection modules. We employed one-month learning window to process the sensors’ observations and to build the underlying behavioural model. Moreover, Table 7 presents the description of the injected abnormal behaviours in all the datasets.

## 5. Results and Discussion

This section presents the obtained results of the conducted experiments. The results are presented with regard to the individual modules of the system and the defined performance metrics.

### 5.1. Learning Module

**Figure 8:**
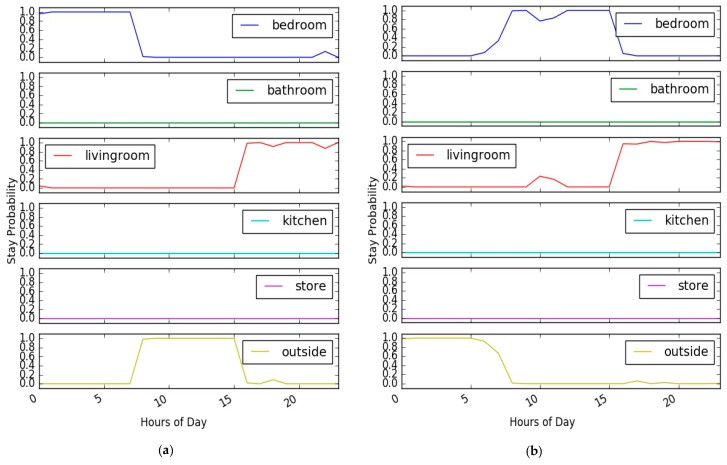
Learned normal behaviour- synthetic datasets. (**a**) Profile A; (**b**) Profile B.

The quality of the learning module is determined by how well it describes the behaviour of the monitored person and the interpretation it provides to better distinguish between normal and abnormal behaviour. Figure 8 illustrates an example of the obtained results of the learning module for a normal day for the two users (profile A and profile B) of the synthetic dataset. The results are presented in a stacked plot to show the comparison of the learned stay probability estimates in all rooms during the hours of the day.

As shown in the figure and based on the given users’ profiles, the three simulated behaviours of the two persons are correctly estimated and high stay probability estimates are given to the rooms where the person usually performs his/her daily normal routine. The learning module was successfully able to learn the normal daily behaviour of the monitored person during each hour of the day, each data point in the figures represents, for the given time of the day, the learned stay probability from the model. As shown, the day is clearly segmented into three segments and high stay probability estimates are assigned to the rooms where the user was active during the hours of the day.

### 5.2. Anomaly Detection Delay (ADD)

**Figure 9:**
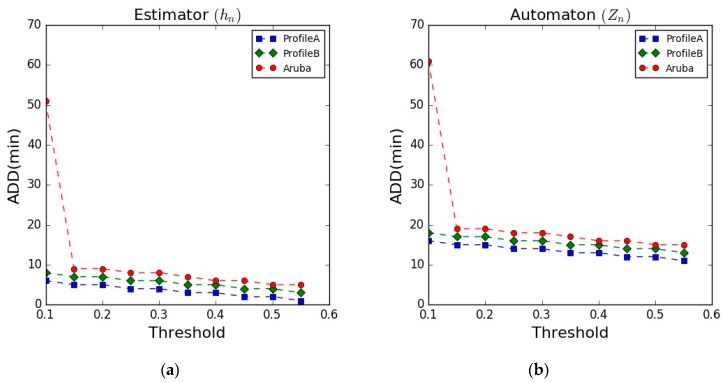
Anomaly Detection Delay (ADD. (**a**) Estimator results; (**b**) Automaton results.

The ADD is used to evaluate the responsiveness of the detection module in terms of the minimum time required to detect an anomaly (abnormal behaviour). Figure 9 illustrates the ADD results on the datasets, showing the results of the estimator and the automaton, respectively. The figure shows the ADD results of detecting the “LessSleeping” abnormal behaviour in all the datasets. In the figure, as the threshold increases, the ADD results slightly decrease in the three datasets. The results of the automaton are higher than the results of the estimator. This is due to the added abnormal timeout (N2) in the automaton.

The ADD results after applying the rule-based classifier into the detection module show a similar trend, however, there was no significant effect of the threshold value on the obtained results. The ADD results after applying the rule-based classifier were reduced to 8, 10, 12 min for the Profile A, Profile B, and the Aruba datasets, respectively. The results were better because this classifier incorporates other dimensions of the model beside the consideration of the “potential abnormal” state from the automaton, as shown in Figure 6. This allows the detection module to detect the abnormal behaviour much faster, considering the other dimensions to confirm the detection. A summary of the obtained results is presented in Table 8.

### 5.3. Anomaly Confirmation Time (ACT).

**Figure 10:**
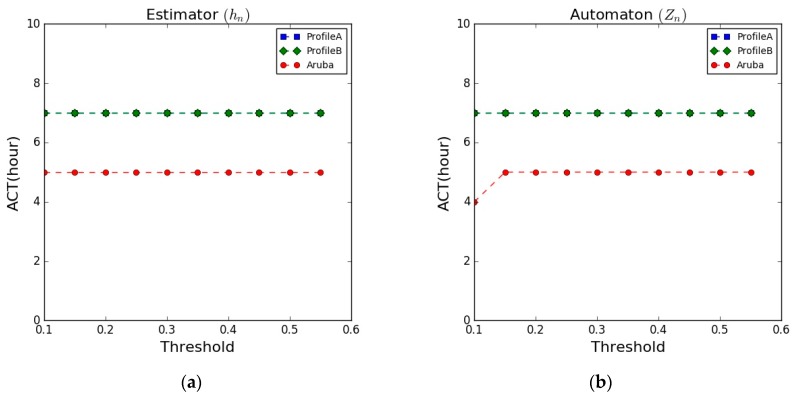
Anomaly Confirmation Time (ACT). (**a**) Estimator results; (**b**) Automaton results.

The ACT aims to evaluate the ability of the developed system in terms of the time taken to confirm the detection of an abnormal behaviour. Figure 10 illustrates the ACT results on the datasets, showing the results of the estimator and the automaton, respectively. 

The figure shows the ACT results of detecting the “LessSleeping” abnormal behaviour in all the datasets. The results of the two user profiles of the synthetic dataset were similar and with no variations as the threshold changes, while the results on the Aruba dataset were lower and also showing steady behaviour as the threshold value changes. The ACT results after applying the rule-based classifier as well did not change significantly as the threshold value changes. The results on the two user profiles of the synthetic data show similar results (more than 7 h) while the results on the Aruba dataset show lower confirmation time (5 h on average). However, the obtained ACT results, in general, showed enough time to confirm the detection of the abnormal behaviour. A summary of the obtained results is presented in Table 8.

### 5.4. Average Number of False Positive Alerts (FP)

**Figure 11:**
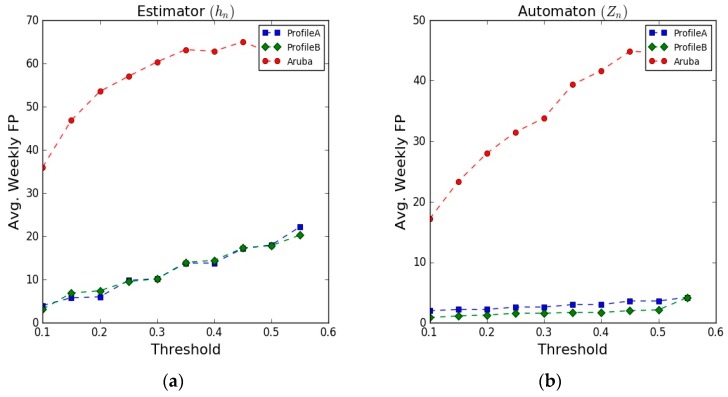
Average of Weekly False Positive alerts. (**a**) Estimator results; (**b**) Automaton results.

Figure 11 illustrates the results of the average weekly false positive alerts detected by the system. The results on the synthetic data show similar behaviour for the two user profiles, as the threshold changes, a low number of false alerts were generated (automaton results), on the other hand, the results on the Aruba dataset show high rates of false alerts. We believe that this high rate of false alerts is due the fact that the Aruba dataset includes days where the monitored person received multiple visits from her relatives during the experiment period. This may affect the accuracy of the learned behavioural model of the monitored person and lead to an increase in the number of false detections.

The results after applying the rule-based classifier show also a low rate of false alerts on the synthetic data and a significant reduction on the false alerts results on the Aruba dataset. The rule-based classifier incorporates some of the other dimensions of the behavioural model to provide the final detection results. This enriches the ability of the detection module to differentiate the abnormal behaviours from the normal routine.

Table 8 presents a summary of the obtained results compared to two approaches from the state of the art [55,56]. These two approaches share some similar objectives with us. The use of simple unobtrusive sensors (PIR) for learning the human behaviour and detecting abnormal behaviour (inactivity periods in this case), the performance evaluation metrics used to assess their systems as well as the dataset used for validation (the Aruba). However, the main distinct difference from our work is that the performance of the developed models in these works depends on prior assumptions about the data distribution of the inactivity periods. In our model, we do not have any prior assumption on the data distributions of the normal movement behaviour of the monitored person, and therefore, our model reduces the modelling settings and provides a more seamless behaviour learning approach.

In the table we present the results of the estimator (h_n_), automaton (Z_n_), and the rule-based classifier (Out_n_), respectively, with respect to the defined performance metrics. The presented results in the table were obtained using 0.25 threshold on the datasets of the “LessSleeping” abnormal behaviour. As shown, our approach outperformed the other approaches and achieved the minimum ADD results (8 min on the synthetic data and 12 min on the Aruba dataset), while the other approaches achieved higher ADD results (23 and 200 min). This gives our approach the capability to detect the occurrence of the abnormal behaviour much faster and allows the recipients of the alarms to react quickly to the abnormal event. The number of weekly false alerts generated by our approach on the synthetic data was low (results of Profile B), while on the Aruba dataset our approach generated higher number of false alerts compared to other approaches. This is relatively due to the multiple visits of the relatives that the monitored resident received during the data collection phase of the Aruba dataset. These visits caused the learned model to be a bit fuzzy, not reflecting exactly the daily routine of the monitored resident. We intend, as future work, to include a more reliable method to eliminate these visits as a pre-processing step before learning the daily behaviour of the monitored person.

### 5.5. Model Adaptation

**Figure 12:**
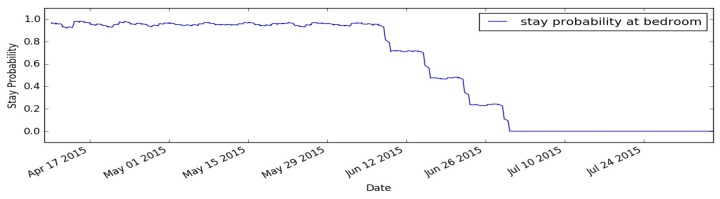
Model Adaptation.

The behaviour model is updated on a weekly basis to incorporate the latest observed behaviour of the monitored person into the model. This allows the model to be adaptive to any behavioural changes that are not necessarily abnormal behaviours. To demonstrate this ability, we performed an experiment in which we merged the two user profiles (profile A and profile B) into a single user profile that simulates a person having a steady behaviour for some time (represented as profile A) and then changes his behaviour and follows different daily profile that extremely varies from the previous one (represented as profile B). To look at the changes during the experiment, we selected the first segment of the day (i.e., hours (0–8]) and focused on the person’s behaviour in the bedroom only. Firstly, the person followed the daily routine of user profile A, which means spending most of the time sleeping in the bedroom during this segment of the day, and then the person changed his/her behaviour and followed the daily routine of the user profile B, which means no sleeping during this segment of the day, and therefore not being detected in the bedroom during this time segment (i.e., hours (0–8]). Figure 12 shows the obtained results of this experiment.

As shown in Figure 12, the learning module was able to estimate the behaviour of the monitored person during the first period of the experiment (profile A), giving high stay probability estimates for staying in the bedroom in this segment of the day. Then the behaviour of the person has changed gradually to new user behaviour (profile B). This happened in clear distinguished four steps that represent the length of the learning window (4-weeks window). As the learning window gets shifted weekly, the model is updated and adapted gradually to the new daily routine of the monitored person. This ability shows how the learning module can adapt its internal behaviour model automatically and perform online and continuous learning of the user’s behaviour.

### 5.6. Classification of Abnormal Behaviour

The classification results of the abnormal behaviours are presented in Table 9. The values in the table illustrate the classification results on the pre-defined abnormal behaviours, as described in Table 7, to show how correctly the detection module was able to identify these abnormal behaviours using the rule-based classifier. The results were obtained using 0.25 threshold in the detection module.

As shown in Table 9, the applied rule-based classifier was able to correctly classify the defined abnormal behaviours with high classification accuracy in most of the cases for the two datasets, except for the “Dead” behaviour on the user Profile B of the synthetic data and the “NotBackHome” behaviour on the Aruba dataset. These abnormal behaviours were a bit tricky and difficult to differentiate. The user Profile B of the synthetic data was designed particularly for a “Nightly” person who usually sleeps during the day and goes outside in midnights. The classification results of the “Dead” behaviour for this profile was mostly misclassified as “LessSleeping”. The presence of the monitored person at any rooms, other than bedroom, during the day would be misclassified and considered as “LessSleeping” behaviour. On the Aruba dataset, the “NotBackHome” behaviour was mostly misclassified as “normal” behaviour. It was difficult for the rule-based classifier to differentiate the time when the monitored person goes outside. These results introduce the need for additional features to be included in the detection module to correctly classify the abnormal behaviours. The classification experiments on the synthetic datasets were repeated three times for each abnormal behaviour and here we present the average of obtained results.

## 6. Challenges and Issues

In this section, we present some of the challenges and issues related to the development of behaviour monitoring systems for Ambient Assisted Living (AAL). These challenges are inspired by prior research studies [5,60,61] and learned while reviewing the related work materials.

### 6.1. Elderly Acceptance

Providing a monitoring service for the elderly poses many challenges. Elderlies, especially unhealthy elderlies, have some limitations in performing tasks. Any service intended for them should pay more attention to the ease of use requirement, avoiding any kinds of complexity or interference that may overwhelm the elderly and reduce their autonomy and independence (ethical issues). This is translated into the selection of sensor modalities used for measuring and monitoring the behaviour, complexity of the system’s settings and assumptions, and service’s transparency and visibility. Social studies [62,63,64] give insights on the real requirements of the elderly and caregivers and also provide solid foundations to support the development of monitoring services for the elderly and help to reduce the lack of confidence in the services.

### 6.2. Data Collection and Representation

The characterization and representation of a behaviour are fundamental for the behaviour monitoring systems. The clear definition of the required behaviour leads to meaningful features extraction and model’s definition and helps to handle behaviours variances. The main challenge here is related to the lack of descriptive knowledge and standards to define the human behaviour in terms of features, heterogeneous sensor data formats, and modelling approaches. Each of the existing systems for behaviour monitoring has their own individual definitions of the behaviour, their own design goals, and modelling approaches, which make the evaluation and comparison of these systems more difficult. Moreover, performing real-world experiments to validate these systems is really difficult. Many studies have been validated using synthetic data [24], or real data collected for short periods of time in very controlled settings. In addition, anomalous behaviour, in general, are rarely happen and we may need a long time of monitoring to detect cases of abnormal behaviour to validate the developed systems.

### 6.3. Privacy

The privacy issue is a major concern for human behaviour monitoring. The type of sensors used for measuring the behaviour is crucial for the user acceptance of the monitoring service. The trade-off between the privacy and the quality of monitoring is always existing, however, the advancement in sensing technologies may provide compromises. For instance, monitoring systems based on camera sensors are not much appreciated, but currently, we have seen considerable advancement in this trend (e.g., the use of low-resolution cameras or images), which may help to address the privacy concerns. Moreover, the right to access the collected data from the monitoring system is also important, to decide the authorised recipients of the monitoring data as well as the allowed data granularity for the recipients.

### 6.4. Cost

The costs of the sensing technologies used for measuring the human behaviour, their installation, calibration, maintenance, and updates costs have to hit the lowest prices to ensure the wide adoption and deployment of the behaviour monitoring systems among a larger sector of people.

### 6.5. Service Quality

The quality of the human behaviour monitoring service is represented in terms of its accuracy, robustness, adaptability, and scalability. The accuracy of detecting and differentiating the behaviour, despite the complexity of monitored behaviours and the different ways of performing the same behaviour, is still a challenge for many behaviour monitoring systems. Moreover, the adaptability of changing behaviour over time as well as the robustness of the monitoring service with respect to context changes (e.g., handling visitors, caregiver visits and pets’ existence at home while monitoring the elderly). The scalability of the monitoring service to cover a larger sector of people is also criteria to evaluate the quality of the service.

## 7. Conclusions

We have presented a system to automatically learn and build an individual model of the daily mobility behaviour of an older adult living alone at home. The system uses location observations collected from low-cost, non-intrusive PIR motion sensors to track the mobility of the monitored person and detect unusual mobility habits. No prior assumptions have to be made about the typical daily behaviour of the monitored person before applying the system. The system can adapt its internal behaviour model to slight shifts in behaviour such as seasonal changes and also to different people having different daily behaviours, such as someone usually sleeping all morning or staying outside the home during the nights. The system provides abnormal alarm notifications in quasi-real-time, in contrast to most of the existing behavioural models and also reduces the rate of wrong detection and false alert notifications. 

As future work, we intend to apply a method to detect visit days to ignore them during the learning of the model, the days when the monitored person receives visits from relatives or friends. A recent work on this topic can be found in [65]. In addition, the consideration of false location detection by the PIR sensors (false detection due to, for instance, heated air or other obstacles) also needs more investigation. A method to exclude the wrong location detection will increase the correctness of the learned model.

## Figures and Tables

**Table 1:** Sensing technologies—Properties comparison.

Property	PIR	Camera	Smartphone	Smartwatch
Location detection	Low	High	High	High
Presence detection	Medium	High	High	High
Tracking	Single user	Multi-users	Multi-users	Multi-users
Resolution	Single bit (on/off)	High	High	High
Cost	Low	High	High	High
Privacy concern	Low	High	High	High
Battery life	High	NA	Medium	Low
Require data processing	Medium	High	High	High
Localization accuracy	Low (room-level)	High	High	High

**Table 2:** Abnormal Behaviours—Descriptions and related health-declines.

Behaviour	Description	Health-Declines
OverSleeping	An extended stay at bedroom; longer than usual.	Mobility problems, strokes.
LessSleeping	Detected motion at one of the rooms, not a bedroom, during the usual sleeping time.	Having sleepless time due to anxiety, depression or may be developing Alzheimer’s diseases.
NotBackHome	Monitored person stayed outside longer than usual.	Having trouble coming back home or get lost or wondering outdoors.
Dead	No movement and long stay at one of the rooms, not bedroom nor outside;	Death.

**Table 3:** Weighted Global Activity Classification.

S_AG_\W_AG_	Very High	High	Medium	Low	Very Low
Very High	√	√	〤	〤	〤
High	√	√	√	〤	〤
Medium	〤	√	√	√	〤
Low	〤	〤	√	√	√
Very Low	〤	〤	〤	√	√

**Table 4:** Datasets Summary.

	Residents	Rooms	Length (Week)	PIR Sensors
Synthetic	1	6	12	6
The Aruba	1	10	25	31

**Table 5:** Users Profiles—Synthetic dataset.

Profile A	Profile B
Profile for a “Morning person” who usually sleeps as most of the people from midnight until morning (hours 0–8) and goes outside during the day (hours 8–16) and also does some domestics activities in the living room in the evening.	Profile for a “Nightly person” who usually goes outside in midnight and sleeps during the morning and afternoon (hours 8–16), and then performs some domestics activities in the living room during the evening (hours 16–23)

**Table 6:** Experimental settings.

Parameter	Value
Learning window size	4 weeks (1 month)
Model update	weekly
Learning window shift	1 week
Detection sampling periods	1 min
Estimator smoothing window size	10 min
Classsifier threshold	[0,1]
Normal timeout (N1)	5 min
Abnormal timeout (N2)	10 min

**Table 7:** Description of the Injected Abnormal Behaviours in the datasets.

Behaviour	Profile A	Profile B	Aruba
OverSleeping	Prolong sleeping at “bedroom” extended to all afternoon, hours (8–19]	Prolong sleeping at “bedroom” extended to all afternoon, hours (16–23]	Prolong sleeping at “Bedroom1” extended to all afternoon up to hour 19
LessSleeping	Being “outside” during sleeping time in hour (0–8]	Being in “kitchen” during sleeping time in hours (8–16]	Being “Outside” in sleeping time in hours (0–6)
NotBackHome	Stay longer “outside” in hours (16–23)	Stay longer “outside” in hours (8–23)	Going “Outside” and not coming back in hours (7–23)
Dead	Long stay at “store” in hours (8–23)	Long stay at “store” in hours (8–23)	Long stay at “office” in hours (7–23)

**Table 8:** Results Summary.

Dataset	ADD (min)			ACT (Hour)					FP (Weekly)				
	h_n_	z_n_	Out_n_	[55]	[56]	h_n_	z_n_	Out_n_	h_n_	z_n_	Out_n_	[55]	[56]
Profile A	4	14	8	-	-	7	7	7	9.8	2.6	1.8	-	-
Profile B	6	16	10	-	-	7	7	7	9.5	1.5	0.7	-	-
Aruba	8	18	12	23	200	5	5	5	57	31	11.2	20.67	1.23

**Table 9:** Classification of abnormal behaviour results.

Dataset	OverSleeping	LessSleeping	NotBackHome	Dead
Profile A	96.26%	97.71%	93.80%	70.69%
Profile B	69.72%	65.37%	74.72%	23.24%
Aruba	81.97%	91.94%	34.71%	83.12%
